# Promoting early goals of care conversations in the CICU with a surprise question-based EHR workflow

**DOI:** 10.1186/s12904-024-01602-4

**Published:** 2024-12-20

**Authors:** Adam Ushpol, Colby Parsons, Sophia Golec, Ritsa Frousios, Surafel Tsega, Anne S. Linker, Maria Ronquillo, Umesh Gidwani

**Affiliations:** 1 Icahn School of Medicine at Mount Sinai, 50 E 98th St, Apt 10J-3, 10029 New York, NY USA; 2 Department of Medicine, NYC Health + Hospitals/Kings County, 451 Clarkson Avenue, 11203 Brooklyn, NY USA; 3 Department of Quality & Safety, NYC Health + Hospitals, 50 Water Street, 16th Floor, 11201 New York, NY USA

**Keywords:** Palliative care, Cardiac intensive care unit, Quality improvement, Outcomes, Electronic health record

## Abstract

**Background:**

The Surprise Question (SQ) - *Would you be surprised if this patient died within the next 6 months?* - is a validated tool for mortality prediction. The Mount Sinai Cardiac Intensive Care Unit (CICU) incorporated the SQ into a novel EHR workflow to identify patients who would benefit from early initiation of Palliative Care (PC).

**Methods:**

Implementation of the SQ proceeded in two steps. During the feasibility pilot (December 2021-March 2022), providers answered the SQ using an *EXCEL* spreadsheet for all CICU patients, without changing other workflows. In April 2022, the CICU launched a new workflow-column built into the Epic patient-list dashboard with the SQ as the backbone. For patients with SQ answers of “NO,” providers were prompted to facilitate and document a goals of care (GOC) conversation. We conducted a retrospective, observational, quasi-experimental study of all admissions to the CICU with SQ = NO between December 2021-September 2022. Clinical data was obtained via EHR query and chart review. We compared the frequency and timing of GOC conversations and the likelihood of redirected GOC (defined as code status change and/or hospice discharge) during the 3-month pilot versus the 6-month implementation period.

**Results:**

195 admissions were included: median [IQR] age 72.0 [61.0, 84.0] years; LOS > 5 days 43.6%; CICU mortality 17.9%. These clinical characteristics were comparable between the pilot (*N* = 57) and implementation (*N* = 138) periods. However, ICU interventions (i.e. mechanical ventilation, renal replacement therapy) were more common among the pilot cohort (52.6% vs. 33.3%, *p* = .015). For the primary outcomes, compared to the pilot period, there was a significantly higher frequency of GOC conversations (61.4% vs. 81.2%, *p* = .004) and GOC conversations < 2 days from CICU admission (40.4% vs. 61.6%, *p* = .007) in the intervention period. There was no difference in the likelihood of redirected GOC towards comfort or no escalation (28.1% vs. 21.0%, *p* = .288).

**Conclusion:**

We facilitated earlier GOC conversations directed to critically ill patients with high mortality risk by integrating the SQ into the EHR.

**Supplementary Information:**

The online version contains supplementary material available at 10.1186/s12904-024-01602-4.

## Introduction

Intensive care at the end-of-life frequently does not align with the goals of patients and their families [1–3]. To address this problem, the Critical Care Choosing Wisely Task Force recommended the following data-driven guideline in 2014: “Do not continue life support for patients at high risk for death or severely impaired functional recovery without offering patients and their families the alternative of care focused entirely on comfort.” [4] The most effective approach to promote compliance with this recommendation remains unclear [5]. Recent evidence indicates that palliative or advance care planning (ACP) services were offered to less than two thirds of patients with the potential to benefit during their hospital stay, highlighting a considerable gap in care delivery [6]. Palliative Care (PC) is stratified into primary and specialty domains. Primary PC, which includes symptom management and goals of care (GOC) discussions, is delivered by front-line providers without subspeciality training in PC. Specialty PC requires clinicians with dedicated training in PC, a limited and increasingly overstretched resource [7]. As the demand for PC outpaces the availability of specialty providers, there has been a concerted push to enhance primary PC to address the unmet need for PC services [8, 9]. 

Contemporary Cardiac Intensive Care Units (CICUs) admit patients with increasingly complex medical conditions with a high prevalence of comorbidities. PC is an essential component of comprehensive care in these settings [10–12]. Heart failure, which affects 6.7 million Americans and accounts for more hospitalizations and greater lengths of stay than any other condition, exemplifies the need for such integrated care [13–15]. However, little has been written about PC interventions in the CICU [11]. In view of this gap, our team developed and implemented a quality improvement initiative to promote GOC conversations in the CICU. We hypothesized that an electronic health record (EHR)-based workflow, designed to identify patients at high-risk of mortality and prompt appropriate follow-up actions, would increase the rate and timeliness of GOC conversations in the CICU.

## Methods

This study evaluates the impact of a quality improvement project designed to increase the rate and timeliness of GOC conversations for patients at high risk for mortality in the CICU via a retrospective, observational, quasi-experimental approach.

Approval for the study was obtained by our local institutional review board. The study satisfied the requirements for waiver of consent.

### Setting

Our project was conducted in the CICU and Cardiac Stepdown Unit (CSDU) at The Mount Sinai Hospital, a large, tertiary academic medical center in New York City. The units have 14 beds and 6 beds, respectively, and specialize in the comprehensive management of patients with complex arrhythmias, advanced heart failure, mechanical circulatory support, and post-cardiac transplantation care. The team includes an attending cardiologist, an attending intensivist, medical trainees (fellows and residents), specialized nurses, respiratory therapists, speech therapists, nutritionists, physical therapists, and a unit-based social worker. Specialty palliative care is available on a consultative basis.

In the CICU and CSDU, GOC conversations were historically initiated at the discretion of the primary attending clinician in collaboration with the unit social worker. These efforts frequently emanated from informal discussions regarding patients’ palliative care needs at daily multidisciplinary rounds; however, no formal structures were in place to identify patients requiring GOC conversations or to initiate them.

### Intervention

Our intervention was conducted between December 2021 and September 2022. It leveraged a validated tool for mortality prediction, the Surprise question (SQ), which asks the primary clinician: “Would you be surprised if this patient died within the next 6 months?” The framework for our intervention was inspired by NYU’s Supportive Care Program, but adapted to our unit’s unique workflows and institution’s EHR infrastructure, and the novel setting of the CICU [16, 17]. 

Implementation of the SQ-based workflow proceeded in two phases. During the feasibility pilot (December 2021-March 2022), the primary clinician answered the SQ using an *Excel* spreadsheet for all CICU patients upon admission, without changing other workflows. The unit’s informal approach to coordinating GOC conversations continued as outlined above. Therefore, this cohort represents the CICU’s baseline for conducting GOC conversations, and is referred to as the pre-intervention group.

**Fig. 1:**
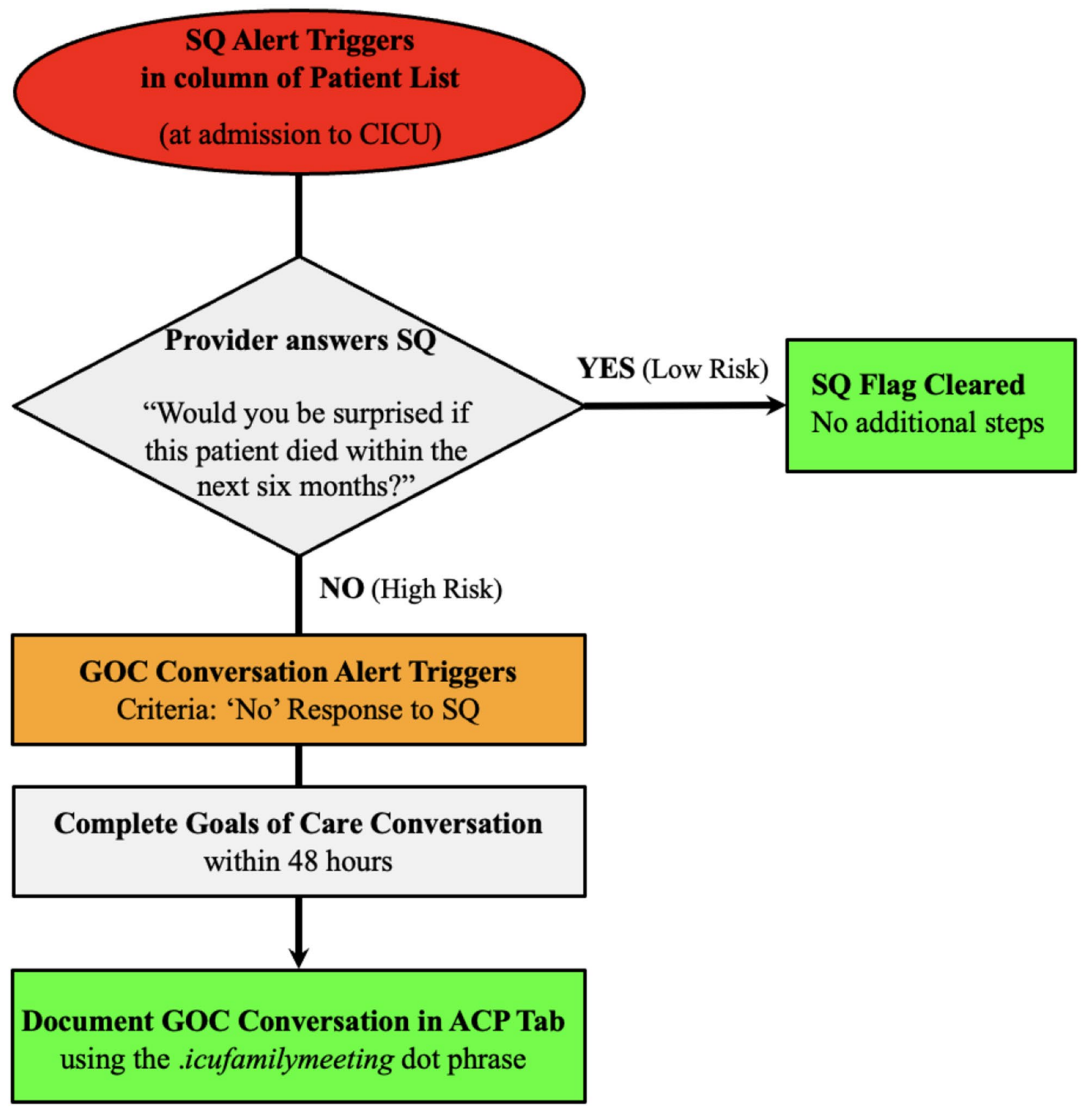
Intervention workflow to promote primary palliative care delivery in the CICU. On all CICU admissions, a red alert populates the patient-list dashboard, requiring the attending clinician to answer the SQ via a pop-up window. 1. If the clinician selects “Yes,” no further action is required beyond standard care. The red alert is automatically cleared and replaced with a green icon. 2. If the clinician selects “No,” a non-interruptive orange alert is activated, indicating a goals-of-care conversation is required. 3. To clear the orange alert, the clinician must complete and document a family meeting using a smartphrase template, which prompts the clinician to note the key elements of the conversation including the shared plan of care. Once complete, the orange alert is automatically cleared and replaced by a green icon

Our hospital utilizes Epic (Madison, WI) for its EHR system. Immediately following the conclusion of the feasibility pilot in March 2022, a new column was built and integrated into the attending patient list dashboard. On admission to the CICU, a red icon would appear within the SQ column on the patient list. This icon served as a non-intrusive alert for the attending to answer the SQ for that particular patient. Double-clicking on the icon would bring up a pop-up window with the SQ. If the attending selected “Yes,” no further action was required beyond standard care, and the red icon was removed. If the attending selected “No,” the red icon was replaced with an orange icon that served as a non-interruptive notification to have a goals of care conversation. To turn the icon green, the attending has to complete and document a family meeting using a note template designed for the project. This template prompted the clinician to note the key elements of the conversation, including the shared plan of care. Once the conversation and note were completed, the orange icon was replaced by a green icon, indicating that the workflow was completed (see Fig. 1; see Supplementary Figs. 1–3). Of note, while other clinicians, such as trainees or consultants, may have participated in or had separate discussions, only the CICU attendings were able to document the GOC conversation as part of the official workflow. Our study tracked these CICU attending-led conversations.

### Study population

All patients admitted to the cardiac intensive care unit during the study period with SQ = “No” were eligible. No patients were excluded.

### Metrics and data sources

A list of all patients with SQ = “No” during the pre-intervention period was obtained via *Excel* spreadsheet. Post-intervention, all patients with SQ = “No” were identified via EHR query. Pre- and post-intervention groups were compared based on demographics, CICU length of stay, rates of ICU interventions (mechanical ventilation, continuous veno-venous hemofiltration (CVVH), hemodialysis, CHF solution), code events and mortality in the CICU. These clinical data were also obtained via EHR query.

Primary outcomes included: (1) the presence, and (2) the timeliness of goals-of-care conversation documentation. For the pre-intervention group, two readers reviewed all notes documented in Epic and counted patient-care team interactions as GOC conversations if the note addressed either: (1) prognosis and/or illness understanding or (2) goals and/or treatment options. These criteria were chosen based on existing literature and the clinical expertise of the research team as core components of shared decision-making [18]. Any discrepancies between the two readers’ assessments were resolved by the broader author group. Usage of the newly created ACP note template that attendings were directed to use was also tracked. We manually reviewed a random selection of these notes to ensure they met the same criteria used to evaluate the pre-intervention notes.

Secondary outcomes included a change in code status, discharge to hospice care, or transfer to the palliative care unit. Together these measures represent GOC redirected towards comfort and non-escalation. These data were collected via EHR query.

### Statistical analysis

Descriptive statistics summarized demographic and clinical characteristics. Continuous variables were presented as medians and interquartile ranges (IQR) and are compared between groups using the Mann-Whitney U test. Categorical variables were reported as frequencies with percentages and are compared between groups with the Chi-square or Fisher exact test as appropriate. To minimize the potential for Type I error, we selected an alpha level of 0.01 for statistical significance. No formal sample size calculation was performed, because the study was conducted as part of a quality improvement project in a real-world clinical setting, where controlling the sample size was inherently challenging. Analyses were performed using SPSS (IBM, Armonk, NY, USA).

## Results

One hundred and ninety-five patients were included. Of these, 58 were in the pre-intervention cohort and 137 were in the post-intervention cohort. Univariate comparison of summary characteristics revealed that the patients did not differ significantly by age, sex, LOS, code events, and CICU mortality. The pre-intervention cohort had higher rates of ICU interventions (52.6% vs. 33.3%, *p* = .015), driven predominantly by higher use of renal replacement therapy (see Table 1).

**Table 1:** Demographics and clinical characteristics of the sample comparing pre-intervention and post-intervention cohorts

	Totals (195)	Pre-Intervention Cohort (57)	Post-Intervention Cohort (138)	*p*-value
Age (median, Q1, Q3)	72.00 (61.00, 84.00)	75.00 (61.50, 84.50)	71.50 (60.00, 83.25)	0.568
Gender (female, %)	83 (42.6%)	24 (42.1%)	59 (42.8%)	0.934
LOS > 5 days (%)	85 (43.6%)	28 (49.1%)	57 (41.3%)	0.317
ICU Intervention (%)	76 (39.0%)	30 (52.6%)	46 (33.3%)	0.015
Mechanical Ventilation	54 (27.7%)	17 (29.8%)	37 (26.8%)	
CVVH	14 (7.2%)	5 (8.8%)	9 (6.5%)	
Hemodialysis	25 (12.8%)	14 (24.6%)	11 (8.0%)	
CHF Solution	13 (6.7%)	9 (15.8%)	4 (2.9%)	
Code Event (%)	3 (1.5%)	2 (3.5%)	1 (0.7%)	0.205
CICU Mortality (%)	35 (17.9%)	13 (22.8%)	22 (15.9%)	0.256

For the primary outcomes, there was a significantly higher frequency in the post-intervention period of total GOC conversations (61.4% vs. 81.2%, *p* = .004) and GOC conversations occurring within 2 days of CICU admission (40.4% vs. 61.6%, *p* = .007). For the secondary measures, there was no difference between pre- and post-intervention rates of GOC redirected towards comfort – i.e. change in code status, discharge to hospice care, or transfer to the palliative care unit (28.1% vs. 21.0%, *p* = .288) (see Table 2).

**Table 2:** Comparison of outcomes between the pre-intervention and post-intervention cohorts

	Totals (195)	Pre-Intervention Cohort (57)	Post-Intervention Cohort (138)	*p*-value
GOCC (%)	147 (75.4%)	35 (61.4%)	112 (81.2%)	0.004
Time to GOCC from CICU Admission (hours, median, Q1, Q3)	26.08 (16.00, 50.64)	38.22 (7.44, 86.25)	25.39 (18.23, 47.83)	0.919
GOCC < 2 days from CICU Admission (%)	102 (52.3%)	23 (40.4%)	85 (61.6%)	0.007
Redirected GOC (%)	45 (23.1%)	16 (28.1%)	29 (21.0%)	0.288
Code Status Change	38 (19.5%)	14 (24.6%)	24 (17.4%)	
Discharge to Hospice	1 (0.5%)	1 (1.8%)	0 (0.0%)	
Discharge to KP6 (Palliative Care Unit)	15 (7.7%)	6 (10.5%)	9 (6.5%)	

## Discussion

Our intervention proved successful in achieving the primary goals of increasing the rate and timeliness of GOC conversations and, ultimately, improving access to primary PC within the CICU setting. The SQ, in our intervention, served to identify those with the greatest need for early PC, and allowed the team to focus resources to accomplish that goal in a timely manner. Our intervention was novel in building the SQ into the EHR and tying the responses to a reminder system to promote specific task completion, reducing variability in provider approaches to GOC discussions. Like other automated alert and reminder systems, this model works by reducing the cognitive burden around planning and initiating GOC [19–21]. Over time, the system of EHR prompts and reminders may secondarily help to encourage habitual adoption or “muscle memory” for providers to engage in prognostication, care planning, and documentation [22, 23]. 

One advantage of the SQ is that it does not use extensive datasets or complex risk stratification algorithms, and instead relies on the clinician’s impression. It is a straightforward, low-cost means to segment a population’s risk of dying and need for primary PC. Our SQ-based model, therefore, proved to be a relatively easy solution to integrate into our existing workflows and has high potential for transferability. While there is mixed evidence about the prognostic value of the SQ, this tool was recently tested on HF patients in the ED and showed promise, with a sensitivity 78.6% of and a negative predictive value of 86.7% [24]. Our CICU admits approximately 1,300 patients each year, with 20–25% screening positive on the SQ. In prior analysis, we found that the SQ identified a subpopulation of patients with high CICU resource utilization and mortality risk [25]. 

Despite an increase in the frequency and timeliness of GOC conversations, there was no significant difference between pre- and post-intervention groups in the proportion of patients discharged to hospice or the PC unit, or with a change in code status. This disconnect between our primary and secondary outcomes is not a shortcoming, because the intervention was not intended to influence families towards any particular care planning decisions. GOC conversations, while crucial for aligning care with patient preferences, do not necessarily lead to changes in care decisions. Families may still choose to pursue full medical interventions following a thorough GOC discussion. We suggest the intervention holds value simply in fostering shared decision-making, and increasing adherence to the evidence-based practice recommendations set forth by the Critical Care Choosing Wisely Task Force [4, 8, 26]. Of note, there was much less dialysis in the post-intervention cohort compared to the pre-intervention cohort. Whether this reflects baseline differences in medical complexity between the two groups or suggests our intervention led to a reduction in the initiation of dialysis in some cases is unclear and beyond the scope of our study.

This study has several limitations. We purposely intended the intervention to align with the unit’s existing workflows. As a result, our intervention may not be transferable for users in other systems. Additionally, the utility of the SQ for risk stratification relies on provider expertise and intuition, and is thus user-dependent. The pre-implementation and post-implementation periods occurred during different months of the year. This temporal difference may have introduced variability in seasonal admission patterns, which could have impacted the frequency of GOC conversations and other outcomes. The study’s primary outcome - GOC conversation completion - was assessed indirectly using EHR documentation. It is possible that elements of advance care planning occurred, but were not documented in ways that were measurable as GOC conversations. This is especially relevant to the pre-intervention cohort, where we had yet to introduce standardized expectations and templates for GOC discussions. Further, while we did assess GOC documentation as a proxy for GOC conversation quality, this may not have fully captured the nuances and quality of the actual conversations. We did not formally educate teams on facilitating high-quality GOC conversations, which may have contributed to the absence of observable differences in secondary outcomes.

Future work should aim to define secondary outcomes that more sensitively capture the benefits and value of more frequent and timely GOC discussions. As is often the case in PC research, it is challenging to quantify the more patient-centered benefits of this work, such as its impact on quality of life and goal-concordant care [27, 28]. Qualitative research could be particularly valuable for gaining insights into how our intervention shapes the patient and family experience. Future investigations should also consider comparing our SQ-based model to alternative risk stratifying modalities.

## Conclusion

Embedding the SQ into an EHR workflow is a viable, cost-effective, easily adoptable approach for leveraging physician expertise to identify patients at high risk for poor clinical outcomes, standardize the process of prognostication, and promote early GOC conversations for patients in the CICU setting.

## Electronic supplementary material

Below is the link to the electronic supplementary material.

Supplementary Material 1

## Data Availability

The datasets used and/or analyzed during the current study are available from the corresponding author upon reasonable request.
