# Supplementary material for: Promoting early goals of care conversations in the CICU with a surprise question-based EHR workflow
Source: BMC Palliat Care. 2024 Dec 20;23:288. doi: 10.1186/s12904-024-01602-4 (PMC11662718; doi:10.1186/s12904-024-01602-4)
Supplement: Supplementary file 1 — Supplementary Material 1 [file 12904_2024_1602_MOESM1_ESM.docx]

**Figure 1: Surprise Question Epic Pop-Up, Provider View**


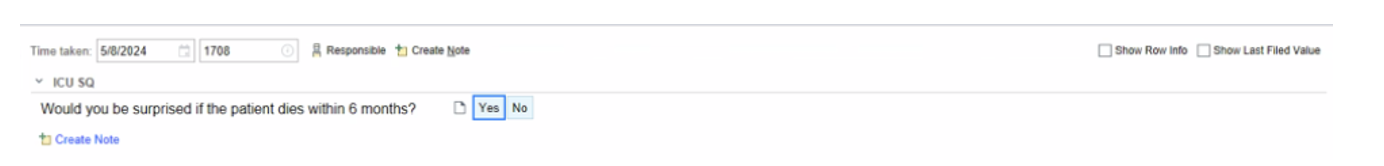


© 2024 Epic Systems Corporation

**Figure 2: Surprise Question Column Workflow Progression, Epic Patient List Dashboard**


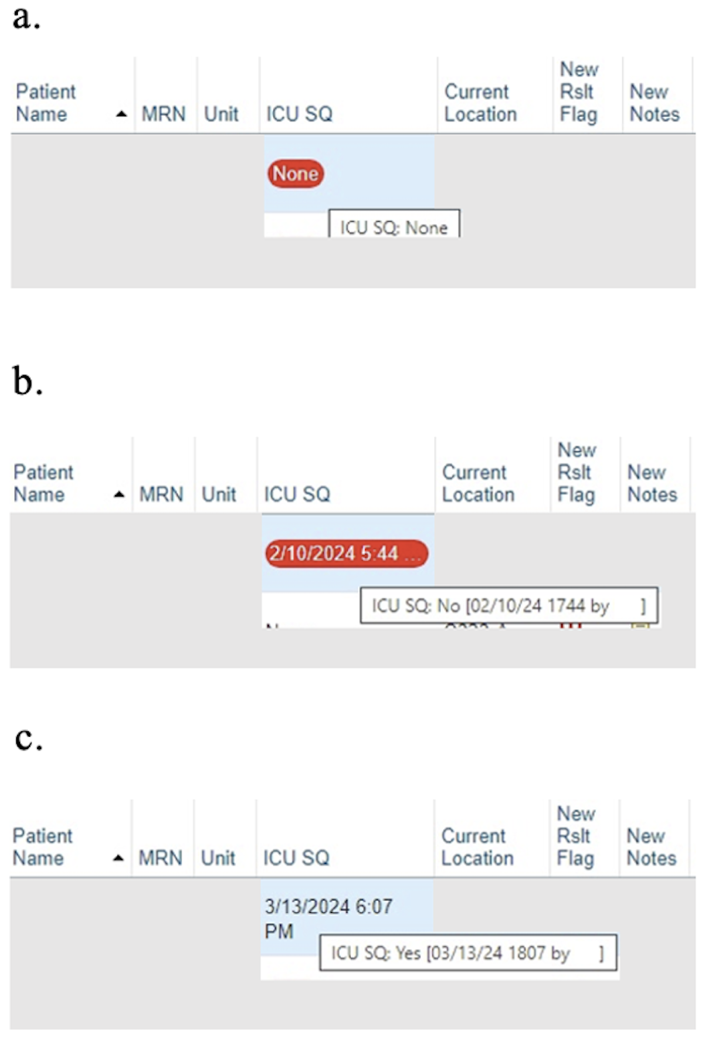


© 2024 Epic Systems Corporation

a. On admission to the CICU, a red icon appears within the SQ column on the patient list dashboard. Double-clicking on the icon brings up a pop-up window with the SQ.

b. If the attending selects SQ = “No,” the red icon is replaced with an orange icon that serves as a non-interruptive notification to have a goals of care conversation. To turn the orange icon green, the attending must complete and document a family meeting using our note template.

c. If the attending selects SQ = “Yes,” no further action is required beyond standard care, and the red icon is removed and replaced with a green icon.

*Not pictured: For patients with SQ = “No,” once the goals of care conversation and note template are completed, the red icon is replaced by a green icon, indicating that the workflow is complete.

**Figure 3: Surprise Question Note Template**


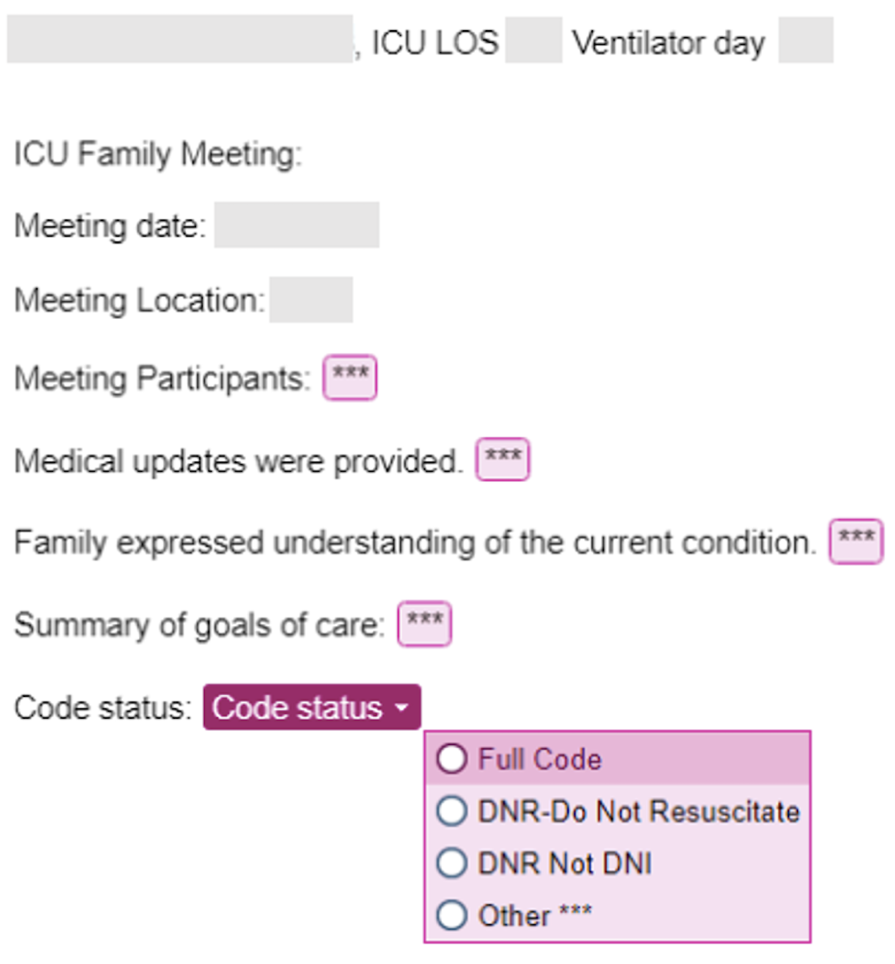


© 2024 Epic Systems Corporation
